# Novel antimicrobial peptides identified in legume plant, *Medicago truncatula*

**DOI:** 10.1128/spectrum.01827-23

**Published:** 2024-01-18

**Authors:** Areej A. Alhhazmi, Sarah S. Alluhibi, Rahaf Alhujaily, Maymona E. Alenazi, Taif L. Aljohani, Al-Anoud T. Al-Jazzar, Ahaad D. Aljabri, Razan Albaqami, Dalal Almutairi, Lujain K. Alhelali, Hibah M. Albasri, Yahya A. Almutawif, Mohammad A. Alturkostani, Abullah Z. Almutairi

**Affiliations:** 1 Medical Laboratories Technology Department, College of Applied Medical Sciences, Taibah University, Medina, Saudi Arabia; 2 Department of Biology, College of Science, Taibah University, Medina, Saudi Arabia; 3 Medical Microbiology Section, King Fahad Hospital, Medina, Saudi Arabia; Universidad Nacional Autonoma de Mexico–Campus Morelos, Cuernavaca, Mexico

**Keywords:** antibiotics, antimicrobial peptide, nodule-specific cysteine-rich peptide, Gram-negative bacteria, *Medicago truncatula*, antimicrobial activity, toxicity, hemolysis

## Abstract

One of the major issues in healthcare today is antibiotic resistance. Antimicrobial peptides (AMPs), a subclass of host defense peptides, have been suggested as a viable solution for the multidrug resistance problem. Legume plants express more than 700 nodule-specific cysteine-rich (NCR) peptides. Three NCR peptides (NCR094, NCR888, and NCR992) were predicted to have antimicrobial activity using *in silico* AMP prediction programs. This study focused on investigating the roles of the NCRs in antimicrobial activity and antibiofilm activity, followed by *in vitro* toxicity profiling. Different variants were synthesized, i.e., mutated and truncated derivatives. The effect on the growth of *Klebsiella pneumoniae* and methicillin-resistant *Staphylococcus aureus* (MRSA) was monitored post-treatment, and survived cells were counted using an *in vitro* and *ex vivo* killing assay. The antibiofilm assay was conducted using subinhibitory concentrations of the NCRs and monitoring *K. pneumoniae* biomass, followed by crystal violet staining. The cytotoxicity profile was evaluated using erythrocyte hemolysis and leukemia (K562) cell line toxicity assays. Out of the NCRs, NCR094 and NCR992 displayed mainly *in vitro* and *ex vivo* bactericidal activity on *K. pneumoniae*. NCR094 wild type (WT) and NCR992 eradicated *K. pneumoniae* at different potency; NCR094 and NCR992 killed *K. pneumoniae* completely at 25 and 50 µM, respectively. However, both peptides in the wild type showed negligible bactericidal effect on MRSA *in vitro* and *ex vivo*. NCR094 and its derivatives relatively retained the antimicrobial activity on *K. pneumoniae in vitro* and *ex vivo*. NCR992 WT lost its antimicrobial activity on *K. pneumoniae ex vivo*, yet the different truncated and mutated variants retained some of the antimicrobial role *ex vivo*. All the different variants of NCR094 had no effect on MRSA *in vitro* and *ex vivo*. Similarly, NCR992’s variants had a negligible bactericidal role on MRSA *in vitro*, yet the truncated variants had a significantly high bactericidal effect on MRSA *ex vivo*. NCR094.3 (cystine replacement variant) and NCR992.1 displayed significant antibiofilm activity more than 90%. NCR992.3 and NCR992.2 displayed more than 50% of antibiofilm activity. All the NCR094 forms had no toxicity, except NCR094.1 (49.38%, SD ± 3.46) and all NCR992 forms (63%–93%), which were above the cutoff (20%). Only NCR992.2 showed low toxicity on K562 (24.8%, SD ± 3.40), yet above the 20% cutoff. This study provided preliminary antimicrobial and safety data for the potential use of these peptides for therapeutical applications.

**IMPORTANCE:**

The discovery of new antibiotics is urgently needed, given the global expansion of antibiotic-resistant bacteria and the rising mortality rate. One of the initial lines of defense against microbial infections is antimicrobial peptides (AMPs). Plants can express hundreds of such AMPs as defensins and defensin-like peptides. The nodule-specific cysteine-rich (NCR) peptides are a class of defensin-like peptides that have evolved in rhizobial–legume symbioses. This study screened the antimicrobial activity of a subset of NCR sequences using online computational AMP prediction algorithms. Two novel NCRs, NCR094 and NCR992, with different variants were identified to exhibit antimicrobial activity with various potency on two problematic pathogens, *K. pneumoniae* and MRSA, using *in vitro* and *ex vivo* killing assays. Yet, one variant, NCR094.3, had no toxicity toward human cells and displayed antibiofilm activity, which make it a promising lead for antimicrobial drug development.

## INTRODUCTION

One of the most health and economic issue worldwide is antibiotic resistance. Three-quarters of these illnesses are caused by resistant bacteria. Challenging infections and high mortality rates have encouraged the scientific community to discover novel antibiotics, particularly from natural resources to combat resistant bacterial strains (1). A group of small molecules such as antimicrobial peptides (AMPs) can be expressed almost by all organisms such as mammalian, insect, amphibian, and microorganisms (2–5). AMPs have no conserved sequences, yet the majority are short, amphiphilic, and highly cationic molecules. Cationic AMPs exhibit antimicrobial activity, targeting various organisms, from bacteria to parasites (6). Antimicrobial activity is not the only activity of these promising antibiotics; numerous studies have shown that these AMPs can exhibit antibiofilm, immune-modulatory, anticancer, anti-inflammatory, and wound-healing activities (7). AMPs can have a potential role in blocking the formation of biofilm, making the bacteria more susceptible to antibiotic treatment, consequently weakening bacterial colonization and pathogenicity.

A novel strategy to fight against antibiotic resistance is to boost the innate immunity response (8). Innate immunity responses are the first line of defense, promptly acting in a non-specific mode (9). Immune enhancement drugs are potent candidates for eradicating bacterial infections effectively (8). Many human peptides, in particular the cationic antimicrobial peptide, which includes defensins and cathelicidin-related antimicrobial peptides LL-37 (10), can excite genes related to innate immunity in human macrophages. Defensins belong to a class of peptides known as small cysteine-rich cationic peptides, which are present across different organisms, such as vertebrate and invertebrate animals, plants, and fungi. They are part of host defense peptides (HDPs) that can act in two different means, direct antimicrobial activity, immune signaling activities, or both (2–5).

Nodule-specific cysteine-rich (NCR) peptides are a large family of peptides that are secreted from rhizobial–legume symbiosis (11). These NCR peptides resemble defensins, small cysteine-rich peptides. NCRs conserve four to six cysteine residues and extremely diverse amino acid sequences, making isoelectric points differ between cationic, neutral, and anionic (12, 13). Synthetic cationic NCRs, particularly NCR247 and NCR335, have antibacterial action against both Gram-positive and Gram-negative bacteria.

In our laboratory, three cationic NCRs (NCR094, NCR888, and NCR992) were predicted to exhibit a potential antimicrobial activity using antimicrobial prediction programs (ClassAMP, AntiBP, and AMPA). The peptides in their wild type and different derivatives, including truncated and cysteine substitution variants, were chemically synthesized. Both defensin and these NCRs are host defense small cationic peptides with conserved cysteine in their sequence. Hence, we hypothesized that the three NCR libraries (NCR094, NCR888, and NCR992) can either have direct antimicrobial effect on bacteria or augment the innate immune response and assist in eradicating bacterial infection. First, the antimicrobial activity of these peptides using standard *in vitro* inhibitory and killing assays against resistant pathogens, particularly methicillin-resistant *Staphylococcus aureus* (MRSA) (Gram-positive bacteria) and *Klebsiella pneumoniae* (Gram-negative bacteria), was performed. Second, the antibiofilm activity of the NCRs and their derivatives was screened by monitoring the reduction in biomass formation on the bottom of a 96-well plate. Finally, the cytotoxicity profile of the NCRs in the wild type (WT) and their derivates was evaluated in two toxicity-based assays: human erythrocyte cell lysis assay and leukemia (K562) cell toxicity assay.

All in all, we identified an undiscovered NCR094.3, exhibiting potential antimicrobial and antibiofilm activities with no toxicity toward human cells. This lead can be an antimicrobial blueprint for further development and optimization in the future.

## MATERIALS AND METHODS

### Strains, chemicals, and growth conditions

Bacterial strains used in the experiment were MRSA ATCC29213 and *K. pneumoniae* ATCC700603. Three NCRs, NCR094 (GenBank# KEH42094.1), NCR992 (GenBank# KEH8992.1), and NCR888 (GenBank# KEH37888.1), and designed derivatives were supplied from GenScript (United States). MRSA and *K. pneumoniae* were streaked for colonies on blood agar plates and incubated at 37°C for 18–24 hrs. MRSA and *K. pneumoniae*’s colonies were cultured in cation-adjusted Mueller–Hinton broth (MHB) media and incubated at 37°C for 18–24 hrs. For the *in vitro* killing assay and the *ex vivo* human killing assay, MRSA and *K. pneumoniae* were cultured on an MHB agar plate for 18 hrs at 37°C.

### *In silico* AMP prediction for NCR sequences using computational analyses

Twenty-two NCR sequences were downloaded from GenBank (Table S1). The basis of the selection was genes encoding NCR secreted peptides. We have conducted a search in the completed genome (https://www.ncbi.nlm.nih.gov/nuccore/APNO00000000.1) in the NCBI nucleotide database. Then, we have randomly downloaded 22 sequences and performed the prediction analysis. NCR’s amino acid sequences were injected into three different AMP prediction programs (ClassAMP, AntiBP, and AMPA) to predict antimicrobial peptides. ClassAMP, a user-friendly web application and available at http://www.bicnirrh.res.in/classamp/, used two different algorithms: random forests (RFs) and support vector machines (SVMs), to forecast the propensity of a peptide sequence that exhibits antimicrobial activity against bacteria, fungi, or virus (14, 15). RFs are clustering methods, built based on multiple independent decision trees that are trained independently on a random subset of data. SVM is a linear model for classification and regression problems that separate data into classes (14, 15). AntiBP is used here in screening potential AMPs among NCR sequences. The algorithm used in AntiBP is based on SVM with focus on the N-terminal and C-terminal of the peptide’s sequences. It predicts only the antibacterial activity of the inquiry sequences. It is accessible at http://www.imtech.res.in/raghava/antibp/ (16). The AMPA algorithm determined the antibacterial sections of each amino acid as well as its bactericidal propensity index. The likelihood of such amino acids being in an AMP sequence is fairly evaluated by the antimicrobial index. AMPA created an antimicrobial profile utilizing a sliding window system and an antimicrobial propensity scale. Amino acids with a low antimicrobial index are given the greatest preference in the AMPA algorithm when creating an AMP. AMP is accessible at http://tcoffee.crg.cat/apps/ampa/do (17, 18). Only short cationic sequences with cysteine residues and sequences projected to have antibacterial action by at least two programs were included in the study to narrow down the pool of candidates for AMP predictions.

### Physical–chemical properties of NCR sequence analyses

Further *in silico* analysis of NCR sequences included the identification of signal peptides and measurements of physicochemical characteristics as hydrophobicity, net charge, cysteine frequency, amino acid composition, and Boman index (protein–protein interaction). The bioinformatic tools for the analysis (https://www.peptide2.com/N_peptide_hydrophobicity_hydrophilicity.php) were fed NCR amino acid sequences.

### Designing new NCR variants

To create various derivatives, NCRs with potential antibacterial action served as a model. In addition to identifying their antimicrobial sections in NCR sequences, the AMPA program was utilized to calculate each amino acid’s bactericidal propensity index. To identify the antimicrobial activity of these variants, the AMPA algorithm was used to produce various types of variants, such as truncation variants of the peptides and replacement of cysteine with serine (17, 18).

### NCR inhibitory and killing assays

A broth microdilution assay in cation-adjusted MHB (MilliporeSigma, United States) was used to determine the minimum inhibitory concentrations (MICs) and the mimium bactericidal concentration (MBCs) of our NCR collection’s NCR094, NCR888, and NCR992. After a final bacterial inoculum of 5 × 10^5^ CFU/mL was set and incubated for an overnight period at 37°C, the MIC of the NCRs that prevented MRSA or *K. pneumoniae* from growing visibly was measured. The MBCs was measured by subculturing the broths used in MIC determination into fresh NCR-free agar plates. The MICs of the NCRs were measured after the NCRs were added to the MRSA or *K. pneumoniae* cultures and dissolved in sterile distilled water.

The whole killing assay was performed as previously described (19). Briefly, freshly drawn peripheral venous whole blood was collected on a heparin tube from healthy adult volunteers. To do this, unchallenged blood with and without NCRs at the MIC (blood from the same individuals) was used. Whole blood was then challenged with MRSA or *K. pneumoniae ex vivo* and incubated for an overnight period at 37°C. Tenfold serial dilution was plated on MH agar media post-18 hrs treatment (19). Killing% was calculated as follows: killing% = CFUs for no treatment − CFUs for NCR treatment ÷ CFUs for NCR treatment × 100. Three independent experiments were performed.

### Antibiofilm assay

*K. pneumoniae* biofilm prevention efficacy of NCRs was assessed in a 96-well platform as reported by reference (20). *K. pneumoniae* was cultivated aerobically for 24 hrs at 37°C. On the second day, *K. pneumoniae* cells were set to optical density (OD600 nm) = 0.02 in RPMI with glutamate medium and 20% glucose in 96-well tissue culture plates. The wells contained *K. pneumoniae* in RPMI with and without NCR added at sub-MIC concentrations (5 µM MIC) of NCRs, and the incubation period was extended by an additional 48 hrs without shaking.

The ability of NCRs to prevent *K. pneumoniae* biofilms was measured using a 96-well format as described by reference (20). *K. pneumoniae* cultures were grown overnight in Luria–Bertani media. On the second day, *K. pneumoniae* (OD600nm  =  0.02) was grown in RPMI glutamate media plus 20% glucose aerobically at 37°C in 96-well tissue culture plates for 24 hrs. The RPMI with and without NCR added at sub-MIC concentrations (5 µM MIC) was added to wells containing *K. pneumoniae* and incubated without shaking for another 48 hrs. Phosphate-buffered saline (PBS: 1× PBS: 10 mM Na_2_HPO_4_, 1.76 mM K_2_HPO_4_, pH 7.4, including 173 mM NaCl, and 2.7 mM KCl) was used to wash the planktonic cells three times. Biomass was fixed with methanol for 20 minutes, stained for the same amount of time with 0.4% crystal violet solution, and then re-solubilized with 33% acetic acid. In a microplate spectrophotometer, the optical density of re-solubilized biomass was determined at 570 nm. The biofilm inhibition percentage was calculated as follows: biofilm inhibition% = optical density at 570 nm for no treatment – optical density at 570 nm ÷ optical density at 570 nm for NCR treatment × 100. Three separate experiments were conducted.

### Human red blood cell hemolysis analysis

With a few minor modifications, red blood cell hemolysis was carried out as previously described (21). On a 96-well microtiter plate, 100, 10, 1, and 0 µM NCRs, NCR094 and NCR992 were placed in each well, and 100 mL of 1× PBS was carried out across the plate. Each well received an equivalent volume of 1% human erythrocytes that had been re-suspended in PBS after being washed once with PBS (5 minutes by centrifugation at 3,000 × *g* to prevent non-specific red blood cell lysis). The plate was then incubated at 37°C for 24 hrs.

As negative and positive controls for hemolysis, respectively, PBS alone and 1% sodium hypochlorite alone were utilized. At a wavelength of 405 nm, hemoglobin release was measured, and the hemolytic capability was expressed as a percentage of the sodium hypochlorite control (100%). The normalization equation normalization = NCR average – negative control average was used to compute the hemolysis percentage. Hemolysis percentage = normalization of NCRs ÷ average of positive control × 100. The hemolysis of 20% and above was considered as toxic to human red blood cells as reported previously (22). The mean values and standard deviation (SD) for each assay were carried out in triplicate and repeated three times.

### Cell culture

Dulbecco’s modified Eagle medium (DMEM) supplemented with 10% fetal bovine serum (FBS) and 1% penicillin (10.000 IU/mL)/streptomycin (50 mg/mL) was used to grow the normal human leukemia cell line (K562) in flasks at 37°C in a humidified environment of 5% CO_2_/air.

#### Cell viability (MTT assay)

K562 cells were cultivated in DMEM with FBS 10% and penicillin–streptomycin 1%, and they were incubated at 37°C with 5% CO_2_ present. Using the MTT assay, the cytotoxicity of the three NCRs was evaluated. K562 cells were grown at a density of 3–4 × 10^4^ cells per well in a 96-well plate and allowed to adhere for 24 hrs. Following the removal of the old medium, the cells were exposed to media with NCRs at varied concentrations (0, 10, and 100 µM). Twenty milliliter MTT (5 mg/mL) was added to each well after the cells had been exposed for 24 hrs, and the cells were then incubated for an additional 4 hrs. Finally, the MTT solution-containing culture media were withdrawn, and 150 mL of dimethyl sulfoxide solvent (DMSO) was used to dissolve the formazan crystals. Using a microplate reader, absorbance was measured at 540 nm. Toxicity percentage = normalization of NCRs ÷ average of positive control × 100. The toxicity of 20% and above was considered as toxic to human cells. Four independent toxicity experiments were computed, and the data were displayed as means and standard deviations.

### Statistical analyses

Each experiment in this work was done in three or two independent replicates. Means and standard deviations were calculated. For comparative analysis, the ANOVA test was performed, followed by *post hoc* or independent *t*-tests for pairwise mean comparisons. To compare paired groups, a paired *t*-test was used. Data <0.05 were considered statistically significant. Excel 16.6 is used for data analysis.

## RESULTS

### *In silico* prediction of NCRs with antimicrobial activities using computational analysis

A collection of 22 NCR sequences from *Medicago truncatula* were extracted from GenBank (Table S1). An *in silico* computational approach was carried out among the 22 NCRs to predict potential peptide sequences with antimicrobial activity. NCR sequences were analyzed using three prediction models: AMPA, AntiBP2, and ClassAMP. Each AMP prediction model employed a different discrimination method to predict the peptide’s antimicrobial activity. NCRs with a bactericidal index more than 0.225 were predicted to have antimicrobial activity based on the AMPA program. NCRs with AMP probability more than 0.5 were forecasted to exhibit antimicrobial activity in ClassAMP, yet AntiBP identified the NCRs as AMP or not. Five of the 22 NCR sequences were selected by all three prediction models, and 12 of them were selected by one prediction model. Five NCRs were predicted to not have antimicrobial activity (Table S1). To further experiment the predicted antimicrobial activity of NCR sequences, only peptides forecasted by the three prediction programs, short peptides (residues 20–35), and those able to be chemically synthesized were included in the study (Table 1). As a result, we narrowed down to novel lead AMP candidates (NCR094, NCR888, and NCR992) for further investigation and experimental testing, since the other two peptides were highly hydrophobic and could be synthesized by the supplier.

**TABLE 1:** The sequence and properties of NCRs and the derivatives used in this study

NCRs	Sequence	Modification	Length	# of cyst	Hydrophobicity%	Cationic charge	Boman index (kcal/mol)	Mean of bactericidal index^*a*^
NCR094	YLKCKTVHDCPKSQVVYRCVGNYCRAVKIRRWNLG	WT	35	4	40	+7.8	2.12	0.226
NCR094.1	YLKCKTVHDCPK	N-12 aa	12	2	25			0.217
NCR094.2	SQVVYRCVGNYCRAVKIRRWNLG	C-22 aa	22	2	36.4			0.19
NCR094.3	YLKSKTVHDSPKSQVVYRSVGNYCR AVKIRRWNLG	C-S	35	0	31.4			0.214
NCR992	MCEFGMIRRCISYKCQCHEAY	WT	21	4	47	+1.25	1.78	0.226
NCR992.1	MCEFGMIRRC	N-10 aa	10	2	40			0.222
NCR992.2	ISYKCQCHEAY	C-11 aa	11	2	18.			0.224
NCR992.3	MSEFGMIRRSISYKSQSHEAY	C-S	21	0	2			0.242
NCR888	MCWPSFKPRCSNGWCVCDKIMP	WT	22	4	50	+1.7	0.94	0.227
NCR888.1	MCWPSFKPRCS	N-11 aa	11	2	28.6			0.224
NCR888.2	NGWCVCDKIMP	C-11 aa	11	2	45.5			0.23
NCR888.3	MSWPSFKPRSSNGWSVSDKIMP	C-S	22	0	45.5			0.246

^*a*^ Bactericidal index more than 0.225 was predicted to have antimicrobial activity based on AMPA program.

### Cysteine composition, hydrophobicity, and cationicity analyses

The three promising NCR peptides (NCR094, NCR888, and NCR992) underwent amino acid composition analysis to understand amino acid preference. The three NCRs had four cysteines in the sequences. NCR888 retained the highest hydrophobicity percentage (50%). NCR094 displayed the highest cationic charge ratio of +7.25 whereas NCR888 held a low cationic charge of +2. NCR992 retained a high percentage of hydrophobicity (46%) but displayed a low cationic charge of +1.25, respectively. For the three promising NCR peptides, the Boman index, which provides details about the peptide’s capacity to interact with biological proteins, was calculated. NCR094 was found to display high protein binding activity with 2.12, followed by NCR092 with a Boman index of 1.78. However, NCR888 had the lowest Boman index with 0.94 (Table 1).

### Designing NCR variants

The likelihood of each amino acid’s antimicrobial activity in the selected peptides was predicted using the AMPA model to acquire additional insight into the sequence function (antimicrobial activity) of the promising NCRs (17, 18). The predicted antimicrobial determining region of NCR094, NCR888, and NCR992 ranges from 11 to 34, 5 to 18, and 8 to 19 residues, respectively (Table 1).

Using AMPA’s results, a synthesized library of three variants of the three wild-type peptides (NCR094, NCR888, and NCR992) included the truncation version of these peptides and substitution alteration in cystine residues. The antimicrobial activity of these derivatives was further forecasted using the AMPA model. The mean bactericidal index calculated by the AMPA algorithm for NCR094 WT was 0.201. In the truncation version, the last C-terminal 22 residues had a lower index (0.19) compared to the WT (0.201). In the other variants (N-terminal 12 residue truncation), Cys-Ser variants had a low index but still higher than the WT (Table 1). NCR992 wild-type and both truncated variants had a low index of 0.22, yet the Cys-Ser variant had the highest index of 0.242. For NCR888, both truncated versions, N-terminal 11 residue truncation and C-terminal residue truncation variants, had a lower index (0.24 and 0.23, respectively) compared to the WT (0.27) and the mutated variant (Cys-Ser) (Table 1). The wild types and the different variants underwent *in vitro* and *ex vivo* experimentation.

### Antimicrobial activity of the NCRs

Three NCRs in their wild type were chemically manufactured (NCR094, NCR888, and NCR992), and their effect on the growth of two resistant bacterial strains, MRSA (Gram-positive) and *K. pneumoniae* (Gram-negative), was monitored post-treatment for 18 hrs by plating on NCR-free agar.

The bacterial cultures of MRSA and *K. pneumoniae* were treated with different concentrations of the synthetic NCRs (MRSA: 25, 12.5, 6.25, 3.125, and 0 µM; *K. pneumoniae*: 50, 25, 12.5, 6.25, 3.125, and 0 µM). The minimum inhibitory (MIC) and killing (MBC) concentrations of *K. pneumoniae*’s NCR094 WT were 12.5 and 25 µM, respectively, and those for *K. pneumoniae*’s NCR992 were 25 and 50 µM, respectively. For MRSA, at the highest concentration tested, 25 µM of NCR094 and NCR992, the killing percentage only reached 6.57% (±1.42) and 20.30% (±2.51), respectively. The tested concentrations of NCR888 did not inhibit or kill MRSA or *K. pneumoniae* post-18 hrs of treatment. Thus, NCR888 in the wild type and the different variants were not included in the downstream assays.

#### The antimicrobial potential of NCR094 in the wild type and different variants

##### NCR094 *in vitro* antimicrobial activity

**Fig 1:**
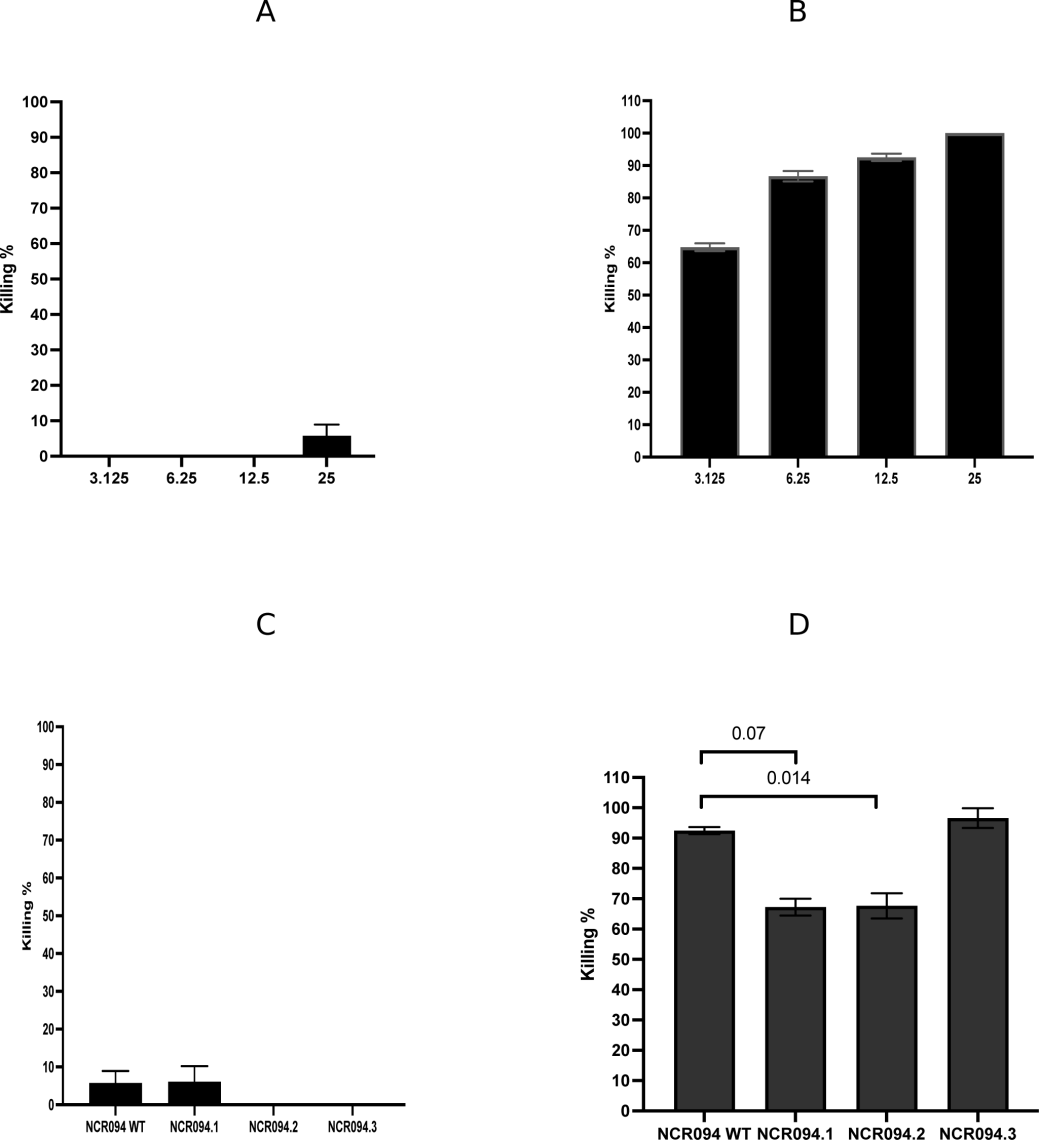
The killing activity of NCR094 in the wild type and the different derivatives on MRSA and *K. pneumoniae in vitro*. (**A**) NCR094 WT at different concentrations, 3.125, 6.25, 12.5, and 25 (µM) on MRSA. (B) NCR094 WT at different concentrations, 3.125, 6.25, 12.5, and 25 on *K. pneumoniae.* (**C**) NCR094 WT and its derivatives at 25 µM on MRSA and (**D**) NCR094 WT and its derivatives at 12.5 µM on *K. pneumoniae* prepared in PBS buffer of bacteria and incubated at 37°C for 18 hrs, and then, the serially diluted bacterial culture was plated on a Mueller–Hinton agar plate at 37°C for 18 hrs. Killing% was calculated. Two independent experiments were done; the results are represented as means of killing% and SDs.

NCR094 WT was the most effective on Gram-negative bacteria, *K. pneumoniae*, more than the Gram-positive bacteria, MRSA, as it eradicated *K. pneumoniae* at low concentrations (25 µM). The highest killing activity reached 100% on *K. pneumoniae* at 25 µM for NCR094, whereas at 25 µM, the killing only reached 5.76% (SD ± 3.17) on MRSA (Fig. 1A and B).

Two derivatives of NCR094 wild type were synthesized, and their ability to kill MRSA and *K. pneumoniae in vitro* was assessed to identify the region(s) responsible for the antibacterial activity. The 12 amino acids from the N-terminal and the 22 amino acids from the C-terminal of the 35 amino acid NCR094 were synthesized. NCR094 WT and its derivatives exhibited almost no killing activity on MRSA *in vitro* (NCR094 WT: 5.76%, SD ± 3.17; NCR094.1: 6.07%, SD ± 4.15; NCR094.2 = 0%; ANOVA, *P*-value =0.133) (Fig. 1C). The wild type of NCR094 showed the highest killing activity on *K. pneumoniae* (92.5%, SD ± 1.15) among the two truncated variants NCR094.1 (NCR094.1; YLKCKTVHDCPK) and NCR094.2 (NCR094.2; QVVYRCVGNYCRAVKIRRWNLG) (ANOVA, *P*-value =0.007; *post hoc* test, *P*-value =0.007 and 0.014, respectively) at 12.5 µM. The N-terminal variant and the C-terminal variant had similar killing effects of approximately 67% (67.2%, SD ± 2.77 and 67.6%, SD ± 4.16, respectively; ANOVA, *P*-value =0.007; *post hoc* test, *P*-value =0.917) at 12.5 µM, suggesting that the two regions in the full-length version of NCR094 were important in the killing activity *in vitro* (Fig. 1D).

A previous study has demonstrated the role of cysteine in the antimicrobial activity of one of the most studied NCRs, NCR247 (23). In this project, four cysteines were replaced by serine in the tested NCRs (NCR094.3; YLKSKTVHDSPKSQVVYRSVGNYCRAVKI). Replacing cystine with serine did not increase the killing activity of NCR094.3 on MRSA, as the killing percentage was only 5.76 (SD ± 3.17) for NCR094 in the wild type and 0 for NCR094.3 (Fig. 1C). However, the cystine replacement with serine retained the killing activity of NCR094 on *K. pneumoniae*, yet it did not significantly augment the NCR094 killing activity (96.6%, SD ± 3.3, ANOVA, *P*-value =0.007, *post hoc* test, *P*-value =0.23) at 12.5 µM, whereas in the wild type, it was 92% (SD ± 1.15) *in vitro* (Fig. 1D).

##### NCR094 *ex vivo* antimicrobial activity

The killing effect of NCRs on MRSA and *K. pneumoniae* was investigated using an *ex vivo* whole blood killing assay as a preliminary screening method to discover peptides with potential immune-boosting activity. The phagocytic cell activity remains existing in the heparinized peripheral human whole blood, which is used in the whole blood killing assay. To determine the amount of CFUs that could be eliminated by the three NCRs in their wild type, MRSA and *K. pneumoniae* were treated with 25 and 12.5 µM, respectively, of NCR094 and NCR992 or without in freshly drawn peripheral human whole blood. The bactericidal ability (killing%) of human whole blood was monitored by counting the CFUs post-18 hrs, and the killing% was calculated.

**Fig 2:**
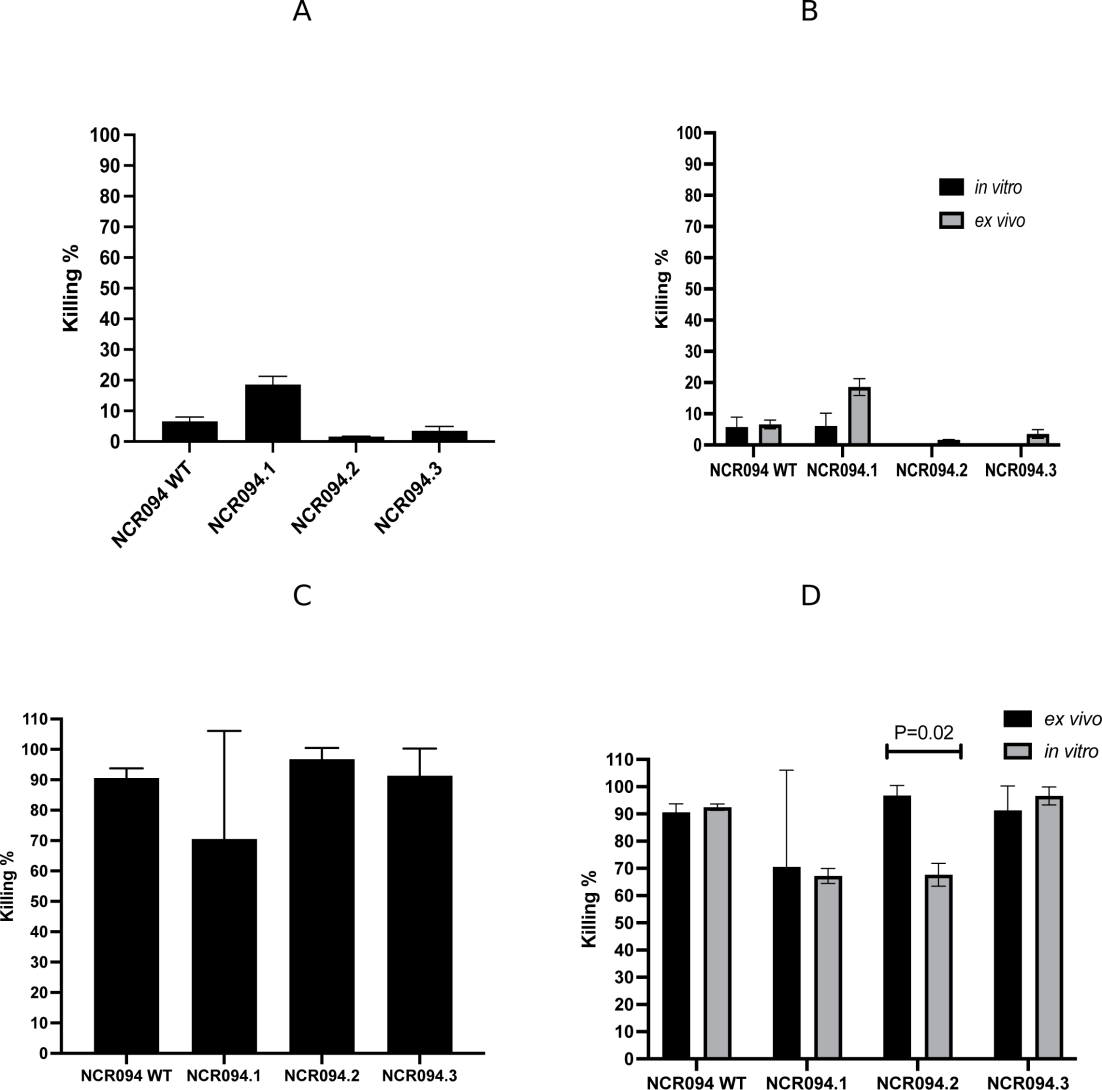
Whole blood killing assay of NCR094 in the wild type and the derivatives, *ex vivo* antimicrobial assay. Whole blood was challenged with two models of bacteria (MRSA and *K. pneumoniae*) followed by NCR094 treatment *ex vivo* for 18 hrs. Post-18 hrs-treated blood was serially diluted and cultured on NCR-free media (MH). CFUs were calculated to estimate the killing percentage. (**A**) Killing% of MRSA *ex vivo*, (**B**) comparison of MRSA between killing% *in vitro* and *ex vivo*, (**C**) killing% of *K. pneumoniae ex vivo*, and (**D**) comparison of *K. pneumoniae* between killing% *in vitro* and *ex vivo.* Three independent experiments were done; the results are represented as means of killing% and SDs.

The wild type of NCR094 had no effect on MRSA *ex vivo* as the killing percentage was 2.76% (SD ± 1.11) at 25 µM (Fig. 2A). NCR094 WT exhibited no effect on MRSA *in vitro* and *ex vivo* (6.57%, SD ± 1.42 and 2.76%, SD ± 1.11, respectively; paired *t*-test, *P*-value =0.184) (Fig. 2B). NCR094 in the wild type exhibited a strong effect on *K. pneumoniae* reaching 90% (SD ± 1.58) *ex vivo* (Fig. 2C). NCR094 in the wild type exhibited a similar killing activity (paired *t*-test, *P*-value =0.512) when *K. pneumoniae* was treated *in vitro* and *ex vivo* (Fig. 2D)*,* yet this did not rule out if the effect was due to the bactericidal activity or innate immunity enhancement or both. All in all, NCR094 in the wild type had no killing effect on the Gram-positive, MRSA, *in vitro* and *ex vivo*.

Using the *ex vivo* whole blood killing assay, NCR094 WT and the truncated variants had similar killing activities on MRSA *in vitro* and *ex vivo* (paired *t*-test, *P*-value =0.184). NCR094.1 exhibited 70% (SD ± 17.7) killing activity on *K. pneumoniae*, while the C-terminal part (NCR094.2) had 96% (SD ± 1.8) killing activity at 12.5 µM (Fig. 2C), suggesting that the C-terminal part appeared to play more role in the killing activity than the N-terminal in the whole blood environment; however, that was not significant (ANOVA, *P*-value =0.564). NCR094.1 had a relatively similar killing activity on *K. pneumoniae in vitro* and *ex vivo* (paired *t*-test, *P*-value =0.907) while NCR094.2 had a higher killing activity *ex vivo* (96%, SD ± 1.8) than *in vitro* (67%, SD ± 4.16) (paired *t*-test, *P*-value =0.0.012) (Fig. 2D).

Additionally, NCR094.3 did not significantly alter the killing of MRSA (NCR094 WT: 6.57%, SD ± 1.48 and NCR094.3: 3.55, SD ± 1.39, independent *t*-test, *P*-value =0.165) (Fig. 2A). Using the *ex vivo* whole blood assay, NCR094.3 had a relatively similar killing activity, 90% (SD ± 1.58) and 92.5% (SD ± 1.15) (paired *t*-test, *P*-value =0.514) on *K. pneumoniae* at 12.5 µM to the wild type of *ex vivo* and *in vitro*, respectively (Fig. 2D).

### The antimicrobial potential of NCR992 in the wild type and different variants

#### NCR992 *in vitro* antimicrobial activity

**Fig 3:**
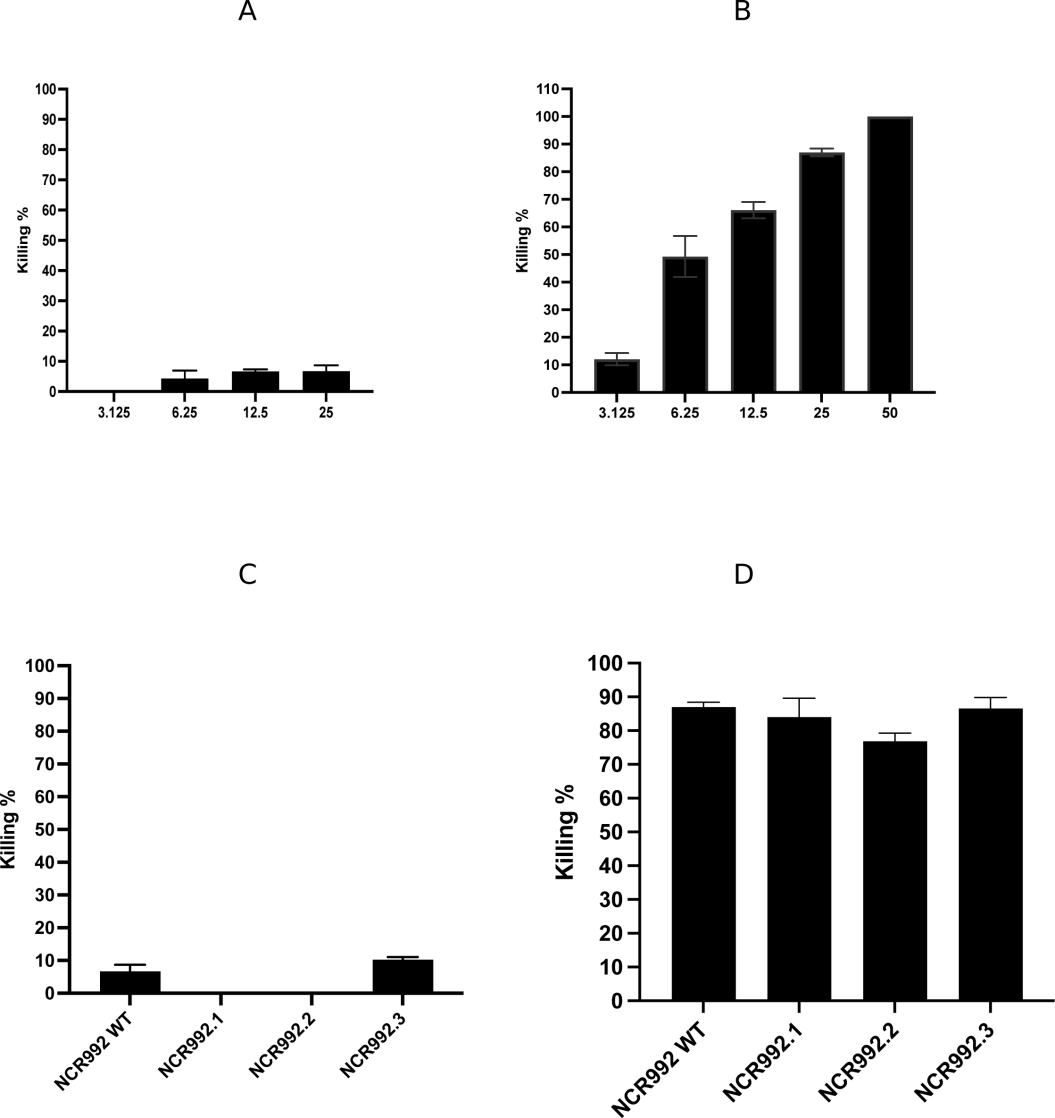
The killing activity of NCR992 in the wild type and the different derivatives on MRSA and *K. pneumoniae in vitro*. (**A**) NCR992 WT at different concentrations, 3.125, 6.25, 12.5, and 25 (µM) on MRSA. (B) NCR992 WT at different concentrations, 3.125, 6.25, 12.5, 25, and 50 (µM) on *K. pneumoniae.* (**C**) NCR992 WT and its derivatives at 25 µM on MRSA and (**D**) NCR992 WT and its derivatives at 12.5 µM on *K. pneumoniae* were prepared in PBS buffer of bacteria and incubated at 37°C for 18 hrs, and then, the serially diluted bacterial culture was plated on a Mueller–Hinton agar plate at 37°C for 18 hrs. Killing% was calculated. Two independent experiments were done; the results are represented as means of killing% and SDs.

At 25 µM of NCR992 WT, it only eradicated 6.73 (SD ± 1.94) of MRSA (Fig. 3A) whereas NCR922 WT at 50 µM killed 100% of *K. pneumoniae* (Fig. 3B). From the 22 amino acid long NCR992 in the wild type, the N-terminal part with 10 amino acids (NCR992.1; MCEFGMIRRC) and the C-terminal part with 11 amino acids (NCR992.2; ISYKCQCHEAY) were manufactured. There was no killing activity exhibited by NCR992 WT and truncated variants on MRSA *in vitro* (NCR992 WT: 6.73%, SD ± 1.94; NCR992.1 and NCR992.2: 0%, ANOVA, *P*-value =0.107) (Fig. 3C). The N-terminal variant, NCR992.1, exhibited 84% (±5.6) killing effect on *K. pneumoniae* while the C-terminal variant, NCR992.2, almost killed 76.9% (±2.4) at 25 µM *in vitro*. The wild type of NCR992 killed almost 87% (±1.4) of *K. pneumoniae* at 25 µM (ANOVA, *P*-value =0.129), indicating that the antibacterial activity *in vitro* was mediated equally by the N- and C-termini as it was by the wild type (Fig. 3D). *In vitro* NCR992.3 had negligible killing activity as it reached only 10.28 (SD ± 0.78), compared to the wild type (6.73%, SD ± 1.94, independent *t*-test, *P-*value = 0.387) (Fig. 3C). For NCR992.3 (MSEFGMIRRSISYKSQSHEAY), the killing activity on *K. pneumoniae* was 86.6%, SD ± 3.2 similar to the WT*,* compared to 87% (SD ± 1.4, ANOVA, *P*-value =0.129) in the wild type at 25 µM *in vitro* (Fig. 3D).

#### NCR992 *ex vivo* antimicrobial activity

**Fig 4:**
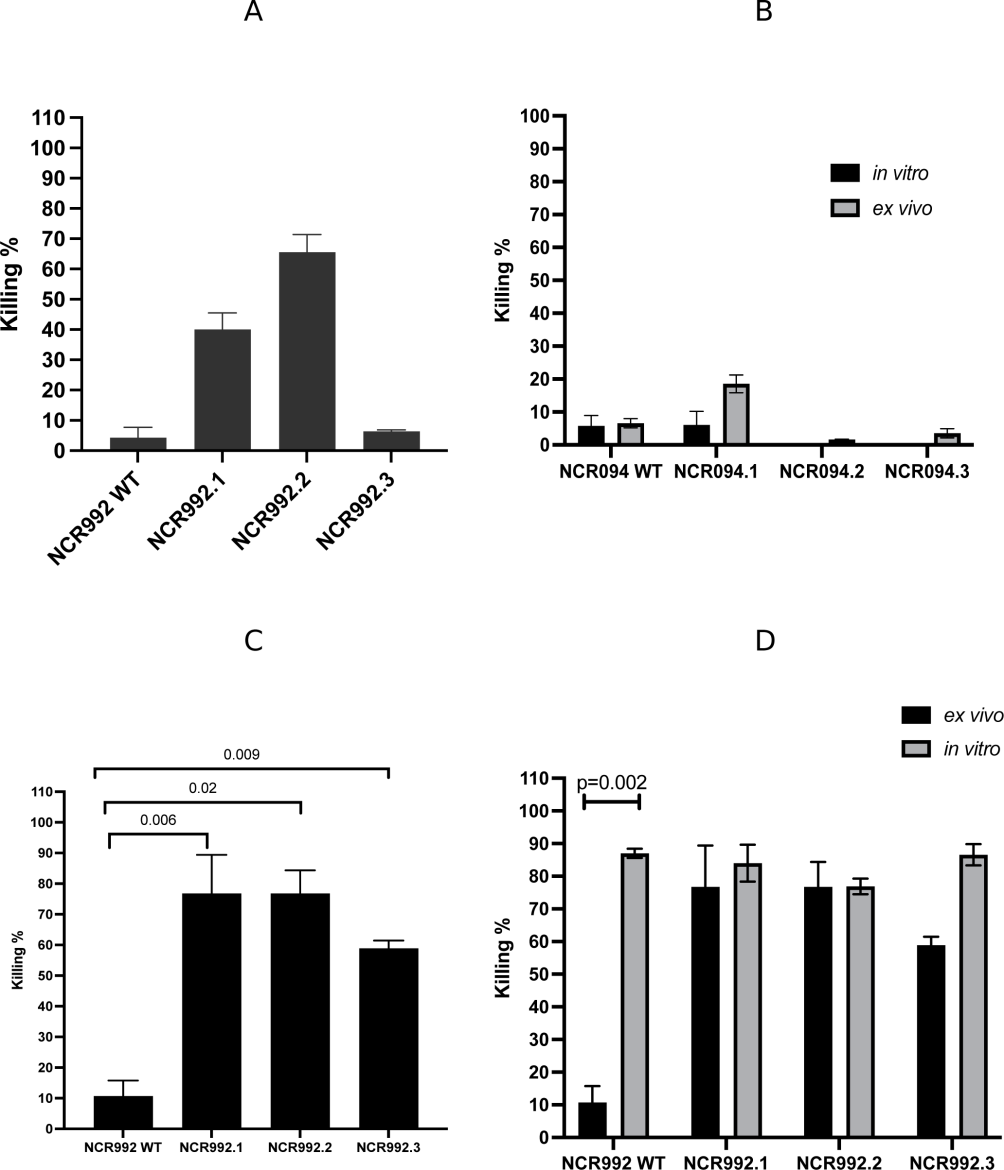
Whole blood killing assay of NCR992 in the wild type and the derivatives, the *ex vivo* antimicrobial assay. Whole blood was challenged with two models of bacteria, MRSA and *K. pneumoniae,* followed by NCR992 treatment *ex vivo* for 18 hrs. Post-18 hrs-treated blood was serially diluted and cultured on NCR-free media (MH). CFUs were calculated to estimate the killing percentage. (**A**) Killing% of MRSA *ex vivo*, (**B**) comparison of MRSA between killing% *in vitro* and *ex vivo*, (**C**) killing% of *K. pneumoniae ex vivo*, and (**D**) comparison of *K. pneumoniae* between killing% *in vitro* and *ex vivo.* Three independent experiments were done; the results are represented as means of killing% and SDs.

The wild type of NCR992 had no effect on MRSA *ex vivo* as the killing percentage was 2.75% (SD ± 1.11) at 25 µM (Fig. 4A). NCR992 WT showed almost no killing effect on MRSA *in vitro* and *ex vivo* as the killing percentage was 4.86% (SD ± 10.71) and 6.87% (SD ± 5.05) (paired *t*-test, *P*-value =0.053) (Fig. 4B). Comparable to the *in vitro* assay*,* NCR992 WT significantly lost its killing activity on *K. pneumoniae ex vivo* (paired *t*-test, *P*-value =0.002) (Fig. 4C and D). NCR992 in the wild type had no killing effect on the Gram-positive MRSA *in vitro* and *ex vivo* and *K. pneumonia*e *ex vivo*.

The killing activity of NCR992 WT on MRSA was 2.75% (±1.11) whereas the truncated variants had a higher killing effect on MRSA (40.09%, SD ± 5.45 and 65.62%, SD ± 5.76, respectively) (ANOVA, *P*-value =0.0003; *post hoc* test, *P*-value ≤0.0001) (Fig. 4A). The wild type showed similar killing activity *in vitro* and *ex vivo* (paired *t*-test, *P*-value =0.238), yet the N- and C-terminal variants (NCR992.1 and NCR992.2) exhibited higher killing activity *ex vivo* than *in vitro* on MRSA (paired *t*-test, *P*-value =0.0.026 and 0.05, respectively) (Fig. 4B). The wild type of NCR992 killed about 10% (SD ± 5.1) of *K. pneumoniae* at 25 µM. The N-terminal variant, NCR992.1, and C-terminal variant, NCR992.2, exhibited 76.8% killing percentage on *K. pneumoniae* at the same concentration *ex vivo* (SD ± 12.6 and ±7.5, respectively, independent *t*-test, *P*-value =1) (Fig. 4C), suggesting that the truncated versions of NCR992 displayed a significantly enhanced killing activity on *K. pneumoniae* compared to the WT in an *ex vivo* whole blood environment (independent *t*-test, *P*-value =0.02 and 0.009, respectively) (Fig. 4C). Only the wild type significantly lost its killing activity *ex vivo*; the two truncated versions (NCR992.1 and NCR992.2) retained the same activity *in vitro* and *ex vivo* (paired *t*-test, *P*-value =0.537 and 0.98, respectively) (Fig. 4C).

The killing activity of NCR992.3 on MRSA had similar negligible effect as the wild type *ex vivo* (NCR992 WT: 2.76%, SD ± 1.11 and NCR992.3: 6.38%, SD ±0.46, independent *t*-test, *P*-value =0.051) (Fig. 4A). There is no significant difference between NCR992.3 effect on MRSA *in vitro* and *ex vivo* (paired *t*-test, *P*-value =0.23). NCR992.3’s killing activity of *K. pneumoniae* significantly enhanced as it reached 58.9% (SD ± 2.52, independent *t*-test, *P*-value =0.0.01) when tested *ex vivo*, compared to 10% (SD ± 5.1) in the wild type (Fig. 4B). The cystine replacement in NCR992.3 boosted the killing activity against the tested Gram-negative bacteria *in vitro* compared to *ex vivo*, yet not significant (paired *t*-test, *P*-value =0.09).

### The antibiofilm activity of the NCRs

**Fig 5:**
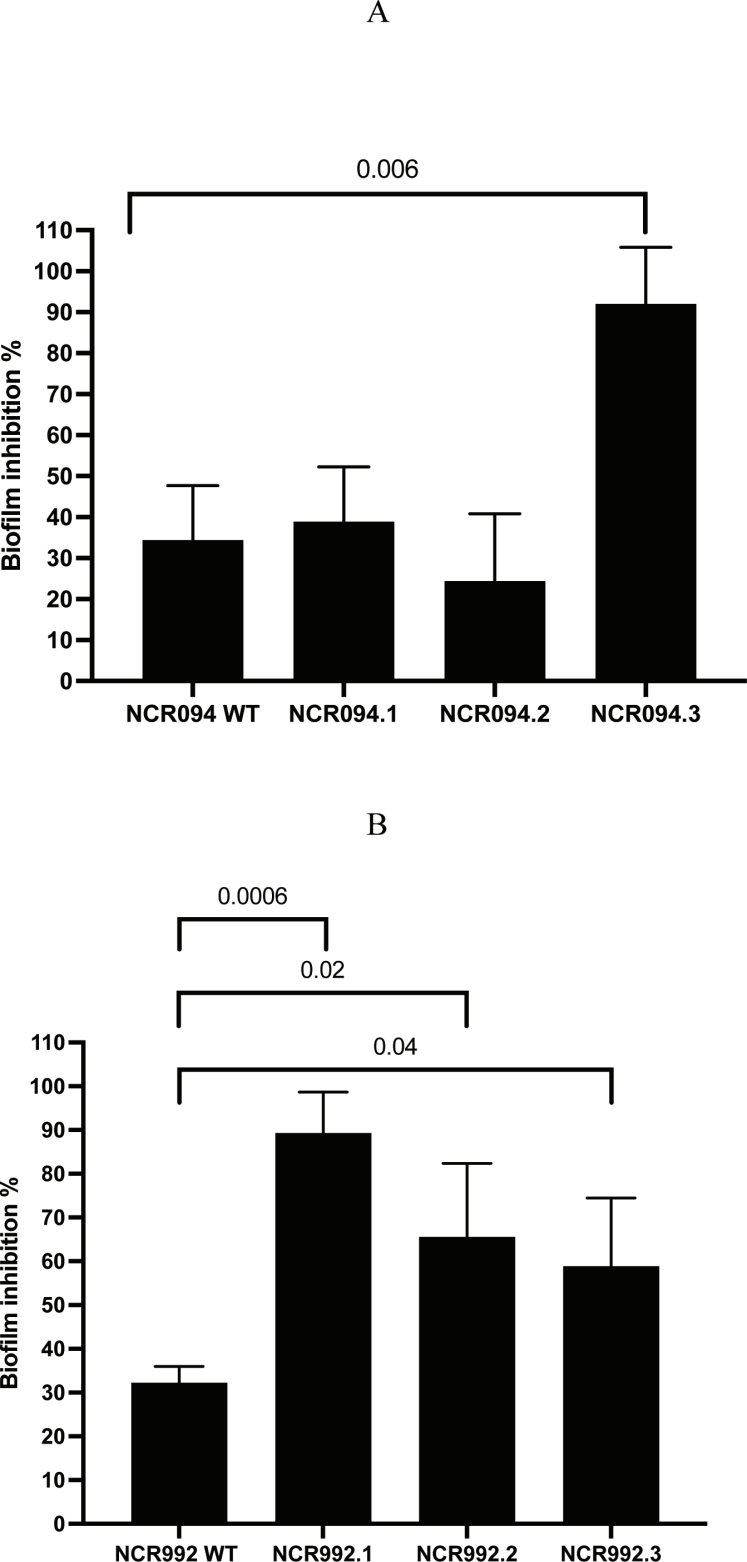
The antibiofilm activity of the NCRs (NCR094 and NCR992) and the derivatives. (**A**) NCR094 including NCR094 WT, NCR094.1, NCR094.2, and NCR094.3 and (**B**) NCR992 including NCR992 WT, NCR992.1, NCR992.2, and NCR992.3. On 96-well microtiter plates, *K. pneumoniae* cells were cultured for 48 hrs at 37°C in the presence of several NCRs at a subinhibitory dose of 5 µM. Crystal violet staining of the adhering biofilm, crystal violet extraction with ethanol/acetone, and measurement of the absorbance at 560 nm were used to study the biofilm formation. Three different experiments' results were calculated as means and standard deviations.

To examine the impact of NCRs on biofilm formation, we used a biofilm formation assay to screen our collection of NCRs. In this assay, 96-well plates were used to analyze the formation of biofilms. To stain the biofilms, 0.4% crystal violet was used (24, 25). The biomass was evaluated for the two NCRs, NCR094 and NCR992 wild type, and the derivatives, and the biofilm inhibition percentage was calculated. Biofilm inhibition by NCR094 and NCR992 in the wild type on *K. pneumoniae* ranged between 32% and 34% (NCR094 WT: 34.42%, SD ± 13.27; NCR992: 32.21%, SD ± 3.75) (Fig. 5A and B). The N- and C-terminal variants (NCR094.1 and NCR094.2) exhibited similar biofilm inhibition percentages to the wild type (NCR094.1: 38.94%, SD ± 13.32 and NCR094.2: 24.41%, SD ± 16.39, independent *t*-test, *P*-value =0.698 and 0.456, respectively). However, cystine replacement augmented the biofilm inhibition by NCR094 (NCR094.3) as it reached 92.08% (SD ± 13.71) (independent *t*-test, *P*-value =0.006) (Fig. 5A). The N- and C-terminal variants and cystine replacement variant of NCR992 had a significantly increased biofilm inhibition percentage compared to the wild type (NCR992 WT: 32.21%, SD ± 3.75; NCR992.1: 89.30%, SD ± 9.35; NCR992.2: 65.61%, SD ± 16.57; NCR992.3: 58.87%, SD ± 15.58, independent *t*-test, *P*-value =0.0006, 0.028, and 0.045, respectively) (Fig. 5B).

All in all, the full version of these peptides had a minor role in inhibiting the biofilm formation of *K. pneumoniae*. The truncated variants of NCR094 showed similar biofilm inhibition activity as the wild type whereas the N- and C-terminal variants of NCR992 showed augmented biofilm inhibition activity compared to the wild type. All the cystine replacement variants of the NCRs has increased biofilm inhibition activity compared to the wild types.

### Toxicity against human cells for the wild-type NCRs

**Fig 6:**
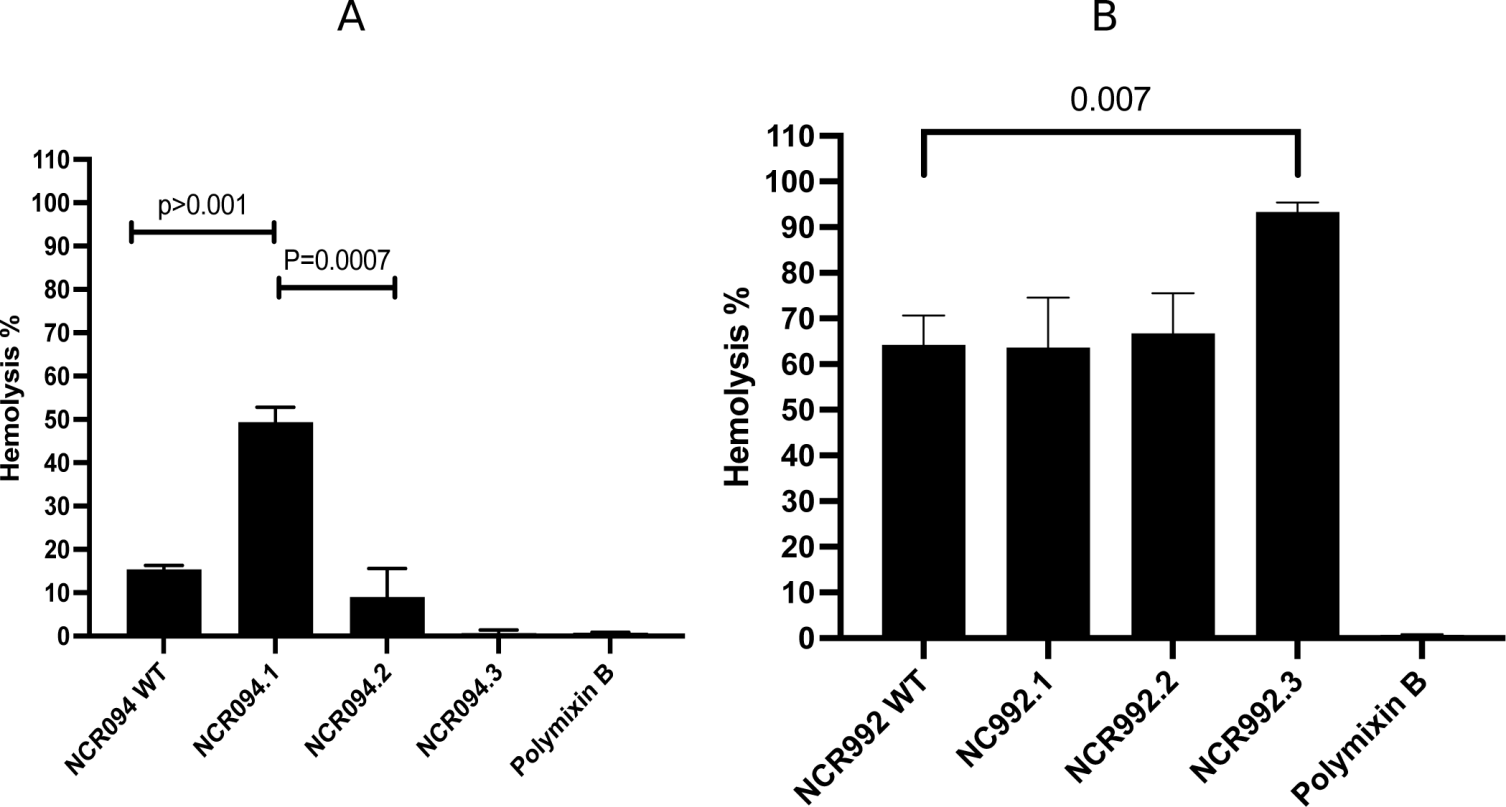
The hemolysis % of the NCRs (NCR094 and NCR992) toward human red blood cells. One percent human red blood cells were treated with 100 µM of the NCRs. (A) NCR094 WT, NCR094.1, NCR094.2, and NCR094.3. (B) NCR992 WT, NCR992.1, NCR992.2, and NCR992 for 16 hrs without shaking. One hundred micromolars of supernatants was transferred into a new 96-well plate, and the absorbance was measured using a plate reader at 405 nm wavelength. Polymyxin B treatment was negative control. The results were represented as average of the % hemolysis and standard deviations from three independent experiments.

The toxicity of NCRs (NCR094 and NCR992) was assessed in their wild type and the derivatives against human erythrocytes. Twenty percentage and above was set as the cutoff to determine whether a peptide is toxic to the human cells. NCR094 in their wild type showed hemolysis below 20% (15.41%, SD ± 0.87). The N-terminal part of NCR094 (NCR094.1) exhibited 49.38% (SD ± 3.46) hemolysis activity, while the C-terminal part (NCR094.2) had 8.99% (SD ± 6.59) toxicity toward erythrocytes (independent *t*-test, *P*-value =0.0007) (Fig. 6A), suggesting that the N-terminal variant by itself exposed a toxicity above 20% (49.38%), compared to both the wild type 15.41% (independent *t*-test, *P*-value >0.001) and the C-terminal variant 8.99%. The replacement of cystine with serine had no significant effect on the hemolysis % activity of NCR094.3 (0.69%, ±0.69) (Fig. 6A). However, NCR992 in the wild type displayed a hemolysis percentage of 64.22% (SD ± 6.46) (Fig. 6A). The N-terminal part (NCR992.1) and the C-terminal variants (NCR992.2) exhibited similar toxicity against human erythrocytes (NCR992.1: 63.67%, SD ± 10.63 and NCR992.2: 66.77%, SD ± 8.13; independent *t*-test, *P*-value =0.94 and 0.70, respectively) at 100 µM concentration (Fig. 6B), indicating that NCR922 in the wild type and the truncated derivatives exhibit toxicity toward human red blood cells. NCR992.3 had more hemolysis activity ranging from 93.34% (SD ± 2.44) at 100 µM than the wild-type activity 64.22% (SD ± 6.46, independent *t*-test, *P*-value = 0.007) (Fig. 6B). To sum up, the peptides that exhibited toxicity toward erythrocytes was NCR992 WT, NCR992.1, NCR992.2, NCR992.3, and NCR094.1.

**Fig 7:**
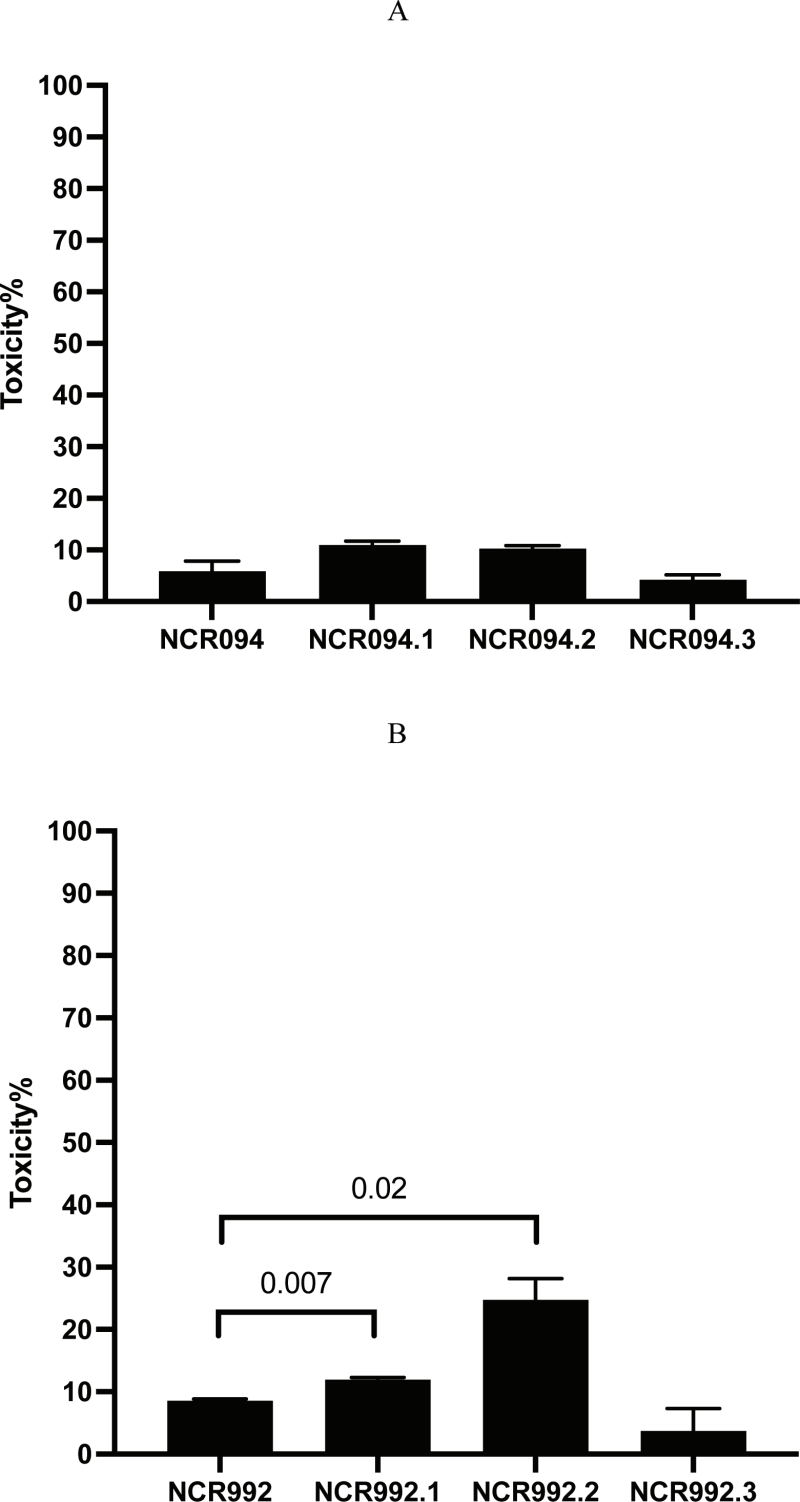
The toxicity % of the NCRs (NCR094 and NCR992) toward leukemia cell line (K562). The cells were treated with 100 µM of the NCRs (A) NCR094 WT, NCR094.1, NCR094.2, and NCR094.3). (B) NCR992 WT, NCR992.1, NCR992.2, and NCR992.3. After a 24-hr exposure, MTT was added, and cells were incubated for 4 hrs. The formazan crystals were dissolved in DMSO. Absorbance was read at 540, and the toxicity% was calculated from four independent experiments, and the results were represented as average of the % toxicity and standard deviations from four independent experiments.

Toxicity toward the leukemia (K562) cell line was assayed, and it was found that all wild types (NCR094 and NCR992) exhibited toxicity below 20%, 5.87% (SD ± 1.97) and 8.54% (SD ± 0.28) (Fig. 7A and B). The NCRs in the truncated version (NCR094.1, NCR094.2) had no toxicity toward leukemia cell lines 10.80% (SD ± 0.73) and 10.07% (SD ± 0.85), similar to the wild type (independent *t*-test, *P*-value =0.0.07 and 0.09, respectively) (Fig. 7A). However, the toxicity of the C-terminal of NCR992 (NCR992.2) exceeded slightly the 20% cutoff toxicity (24.8%, SD ± 3.40, independent *t*-test, *P*-value =0.02) whereas the N-terminal NCR992.1 had toxicity below the cutoff 20% (11.02%; SD ± 0.32, independent *t*-test, *P*-value =0.007) (Fig. 7B). In K562 cells, the change of cystine to serine (NCR094.3 and NCR992.3) had no toxicity, similar to the wild type (4.24%, SD ± 0.94, independent *t*-test, *P*-value =0.40; 6.25%, SD ± 1.2, independent *t*-test, *P*-value =0.019) (Fig. 7A and B).

NCRs were assayed toward human erythrocyte and leukemia cells ranging from 100 to 1 µM to determine whether they have toxicity in a dose-dependent manner. NCR094.1, NCR922 WT, NCR992.1, NCR992.2, and NCR992.3’s toxicity against red blood cells was displayed in a dose-dependent manner ranging approximately from 93% for 100 µM to 4% for 1 µM (Fig. S1A through C). NCR992.2 showed toxicity in a dose-dependent manner ranging from 24.8% (SD ± 3.4) for 100 µM to 9.98% (SD ± 0.82) for 1 µM (Fig. S2).

## DISCUSSION

The global threat to public health posed by antimicrobial resistance is expanding rapidly. Research has shown that bacteria can develop resistance to almost all drugs (26). It is crucial that new antimicrobial medications be discovered. AMPs, which have broad-spectrum activity against multidrug-resistant microbes, have been proposed as the next generation of antibiotics (27). The legume plant *M. truncatula* produces more than 700 distinct NCR peptides in its roots (28). Two NCRs, NCR247 and NCR335, were found to have antibacterial action against a variety of pathogens in earlier research (28, 29). Both NCR247 and NCR335 have the capacity to damage bacterial membranes both inside and externally, which would cause the membrane potential to be lost (30, 31). A recent research found that heme and its bound iron can be sequestered by NCR247, leading to an iron-deprived environment, suggesting a possible antimicrobial mechanism to kill microbes (32). In this study, an *in silico* computational approach was carried out among a collection of 22 mature NCR sequences from *M. truncatula* to predict peptides with promising antimicrobial activity.

Three NCR peptides (NCR094, NCR992, and NCR888) were predicted to have antimicrobial activity and chemically synthesized in this study. The three NCRs are cationic, are short in length, exhibit high hydrophobicity, and can form four possible intramolecular disulfide bonds in the oxidized forms due to four cystines in their sequence. Although having been adapted to perform symbiotic roles, the NCR peptide sequences share structural similarities with the defensin class of antibacterial peptides. These features coupled with the *in silico* prediction rationalized our hypothesis that these peptides can have potent potential antimicrobial activity. The two peptides NCR094 and NCR992 exhibited different potency to destroy one of the most challenging bacteria that cause incurable diseases. NCR094 eradicated *K. pneumoniae* at a lower concentration (25 µM) than NCR992 (50 µM). NCR094 conserved its antimicrobial activity upon testing *in vitro* and *ex vivo*, yet NCR992 lost the antimicrobial activity when tested *ex vivo*. However, testing both NCRs in the wild type with another challenging Gram-positive bacteria, MRSA, had negligible antimicrobial effect *in vitro* and *ex vivo*.

Each of these peptides has its characteristics in canonicity, hydrophobicity, nature of amino acid composition, and formation of disulfide bonds. Hydrophobicity, cationic charge, and secondary structure are the main physicochemical driving forces shaping the interactions between AMPs and their targets (33). A combination of high hydrophobicity and cationicity may improve peptide antimicrobial activity. Consequently, the peptide sequence and length alteration can change its configuration and tune its role in antimicrobial activity. It has been found that the most studied NCRs, NCR247, and different variants have shown various antimicrobial activity levels due to variations in the proper folding of NCR247, which have the main effect on the antimicrobial activity of this peptide (34).

One of the limiting factors of using peptide as therapeutic drugs is poor stability and high degradation rate by a wide range of enzymes. A study by reference (35) concluded that the proteolytic stability of a collection of therapeutic peptides vary depending on the tested matrices, such as serum, plasma, or whole blood. The tested matrix here was whole blood with EDTA, which is a metal ion-chelation factor to block the coagulation process. This changed the proteases and metabolite profiles of the blood and could modify the test peptide stability when antimicrobial activity was assessed. This variation between NCR094 and NCR992 basically depends on peptide-specific sequence properties (35). Both peptides when fed into https://web.expasy.org/cgi-bin/peptide_cutter/peptidecutter.pl had multiple possible cleavage sites that are prone to degradation. It is possible that NCR992 degraded in a form and deactivated its antimicrobial activity seen *in vitro*. However, NCR094 degraded into version(s) that could have reserved the antimicrobial activity. Another possible explanation is that the active site of the peptide was masked by the metabolites present in the whole blood. It has been reported in reference (36) that the presence of at least one cystine is sufficient for the bactericidal activity of NCR247, yet the formation of the double sulfide bond is not essential for the bactericidal activity. The stability of our NCRs can be formed by the formation of double sulfide bonds and through the folding in an oxidative environment. Hence, the stability of the tested NCRs is warranted to be investigated in future directions.

The variation observed in the antimicrobial activity of both NCRs with MRSA and *K. pneumoniae* can be explained due to differences in the surface structure and intracellular targets. The outer cell envelopes vary between the two groups, although the inner or cytoplasmic membranes are comparable. In Gram-positive bacteria, a layer of crosslinked peptidoglycan is decorated with negatively charged teichoic acid surrounding the cytoplasmic membrane, forming a thick matrix. This layer maintains the rigidity of the bacterial cell, which challenges the penetration of peptides into the peptidoglycan layers (37). In contrast, the peptidoglycan layer in Gram-negative bacteria is a thin layer of crosslinked peptidoglycan, which is decorated with an extra outer membrane layer with many negatively charged phosphate groups. In Gram-negative bacteria, AMPs disrupt both outer and cytoplasmic membranes, leading to disrupting the outer membrane and eventually killing bacterial cells (34).

We further investigated the bactericidal properties of different derivatives of these peptides using *in vitro* and *ex vivo* assays. Examining the antimicrobial activity of NCR094’s synthesized N- and C-terminal halves found that both variants showed negligible antimicrobial activity as the wild type on MRSA *in vitro* and *ex vivo*. Assaying the antimicrobial activity of the truncated variants on *K. pneumoniae* revealed that the N and C versions (NCR094.1 and NCR094.2) had a similar killing impact *in vitro*, albeit less than the full version (NCR094 WT). However, the *ex vivo* killing assay showed that the C-terminal variant (NCR094.2) had a higher killing effect than the N-terminal variant (NCR094.1) and the WT *in vitro* and the wild type *ex vivo*. NCR992’s truncated variants (NCR992.1 and NCR992.2) displayed similar antimicrobial activity to the wild type on MRSA *in vitro*, yet higher than the wild type *ex vivo*. For NCR992, the bactericidal effect of NCR992.1 and NCR992.2 had the same effect as the WT *in vitro*. The wild type lost its antimicrobial activity when tested *ex vivo*; however, the different truncated versions (NCR992.1 and NCR992.2) retained the antimicrobial activity as the WT and the two truncated variants *in vitro*. It appeared that NCR094 had no antimicrobial role on the tested Gram-positive bacteria, MRSA, *in vitro* and *ex vivo*. However, the active variant NCR094 and NCR992 configuration in the whole blood environment allowed the peptides with the truncated variants to access the Gram-negative bacteria, *K. pneumoniae*, and efficiently killed the bacteria or enhanced the peptide stability and accumulation to the toxic concentration. The bactericidal activity of NCR992 in the wild type was negligible *in vitro* and *ex vivo*, yet the truncated version showed higher activity *ex vivo* on MRSA and *K. pneumoniae*. It is plausible that the whole blood creates an environment that enhances the activity of NCR992 truncated variants to eradicate the bacteria.

The antimicrobial study of variants with serine instead of cystine indicated that the replacement reserved the antimicrobial activity of NCR094 *in vitro* or *ex vivo* on *K. pneumoniae*, yet it augmented the antimicrobial activity of NCR992 *ex vivo*. Replacement of cysteine in NCR094.3 or NCR992.3 did not increase the antimicrobial activity on MRSA or *K. pneumoniae* in *in vitro* or *ex vivo* settings. Results indicated that, in contrast to NCR247, where one or more cysteines were crucial for the bactericidal activity against *Sinorhizobium meliloti* (36), one or more cysteine residues hindered the killing effect of NCR992 on *K. pneumoniae ex vivo*. The comparison between the antimicrobial activity of NCR094 in the wild type and the truncated variants indicated the importance of the full length of the peptide, yet the replacement of cystine with serine showed similar activity to the wild type. This suggest that at least the middle part of the peptide of the two truncated variants (SQVVYRSVGN) is essential for the antimicrobial activity *in vitro*. This can be further investigated in the future. In order to shape the interactions between peptides and their targets, hydrophobicity, cationic charge, and secondary structure have been identified to be the primary physicochemical driving forces (38). It has been discovered that variations in NCR247’s folding had a major impact on the peptide’s antimicrobial activity (36)

Peptides that are considered toxic in the study was any peptide that showed 20% or above toxicity to human cells as reported previously (22). NCR094, NCR094.2, and its cystine substitution variant (NCR094.3) showed no toxicity toward the red blood and leukemia cells. The truncated version of NCR094 indicated that the N-terminal part (NCR094.1; YLKCKTVHDCPK) displayed toxicity toward red blood cells 49.38% (SD ± 3.46) unlike the wild type. No toxicity was detected toward the leukemia cell line by the truncated versions. NCR992 and its variants displayed toxicity against red blood cells, yet only the C-terminal truncated variant (NCR993.2) showed slight toxicity toward leukemia cell lines. Regardless of the toxicity model, our NCRs’ derivatives (NCR094.1, NCR992.1, NCR992.2, and NCR992.3) may have enabled the peptide to fold in a certain secondary structure, exposing more hydrophobic residues to interact with red blood cells and the cytoplasmic membrane of leukemia cells and cause toxicity. The sequence of the peptide contains hydrophobic and charged domains that enable the peptide to fold into secondary structures like helixes or sheets (39). Yet, the higher cytotoxicity of cationic peptides has been linked to the hydrophobic percentage. Several hydrophobic and charged residues have been shown to help the peptide form a secondary structure (40), changing the toxicity profile of AMPs. The effective action of NCRs, particularly the extensively researched NCR247 *in vitro*, depends on the cysteine residues (23). The S-S bond configuration is essential to shape the NCR secondary structure; the presence of cystine or replacement by serine can have an effect or not to mask the functional domain in the peptide to present activity as demonstrated with NCR247 (23). The most common initial toxicity assessment method is red blood cell hemolysis, which is commonly used as a model for mammalian cell membranes (41). Due to the simplicity of isolating erythrocytes, this model is user-friendly, making this test a flexible tool for initial toxicity assessment (42). However, a further standardized technique to determine toxicity is by performing viability assays against different types of human cell lines. However, hemolysis has been associated with numerous limitations. Two factors that can affect the reliability of the human erythrocyte hemolysis assay are human erythrocyte source and washing techniques with different methods and buffers (43). In our study, we tested the toxicity of our NCR collection against only one cell line, leukemia cell line (K562), and only one NCR992.1 showed slight toxicity, unlike the hemolysis that identified more than one peptide with a toxicity effect. The outcome can be explained by the cell’s greater resilience or more of NCR inactivation due to the addition of fetal bovine serum (43). Yet, in our experiment, fetal bovine serum was present only to cultivate the cells, but not with the NCR treatment.

This study marks the pioneering effort in delineating potential antimicrobial peptides for possible therapeutical applications. This study aims to investigate the bactericidal properties of these peptides using *in vitro* and *ex vivo* assays, which will give a closer snapshot to biological functionality. The study characterized the toxicity profile of the peptides and different variants. However, this study only characterized the antimicrobial activity for two challenging pathogens, MRSA and *K. pneumoniae,* using *in vitro* and *ex vivo* assays. Hence, the generalizability of the findings may be limited due to the limited number of pathogens tested. A more comprehensive profiling including other problematic pathogens will be considered in the future. Assaying these peptides in animal models will increase the reliability and the potential use of these peptides in therapeutical applications, which will be warranted to be discovered in future directions.

In conclusion, preventing and treating infections brought on by opportunistic pathogens like MRSA and *K. pneumoniae* continue to be difficult, in large part because the host defense of the susceptible person is insufficient to fend off serious illness and that the microbe is frequently multidrug-resistant. NCR peptides are one of the promising new treatment drugs. We identified NCR094.3 with potential antimicrobial and antibiofilm activity against a challenging bacterium, *K. pneumoniae*, and no toxicity on human cells. In addition, it is worthy to explore further functional studies such as the immune modulatory activity of these peptides and investigate its antimicrobial activity in animal models.

## SUPPLEMENTAL MATERIAL

Figures S1 and S2 spectrum.01827-23-s0001.pdf Fig. S1 (Dose-dependent hemolysis assay for the NCR collection used in this study) and Fig. S2 (Dose-dependent toxicity assay for NCR collection used in this study toward Leukemia cell line [K562]).

Table S1 spectrum.01827-23-s0002.CSV List of the twenty-two NCRs included in the study and antimicrobial peptide (AMP) prediction program results.

ASM does not own the copyrights to Supplemental Material that may be linked to, or accessed through, an article. The authors have granted ASM a non-exclusive, world-wide license to publish the Supplemental Material files. Please contact the corresponding author directly for reuse.
