# Supplementary material for: Novel antimicrobial peptides identified in legume plant, Medicago truncatula
Source: Microbiol Spectr. 2024 Jan 18;12(2):e01827-23. doi: 10.1128/spectrum.01827-23 (PMC10845954; doi:10.1128/spectrum.01827-23)
Supplement: Figures S1 and S2 — Fig. S1 (Dose-dependent hemolysis assay for the NCR collection used in this study) and Fig. S2 (Dose-dependent toxicity assay for NCR collection used in this study toward Leukemia cell line [K562]). [file spectrum.01827-23-s0001.pdf]

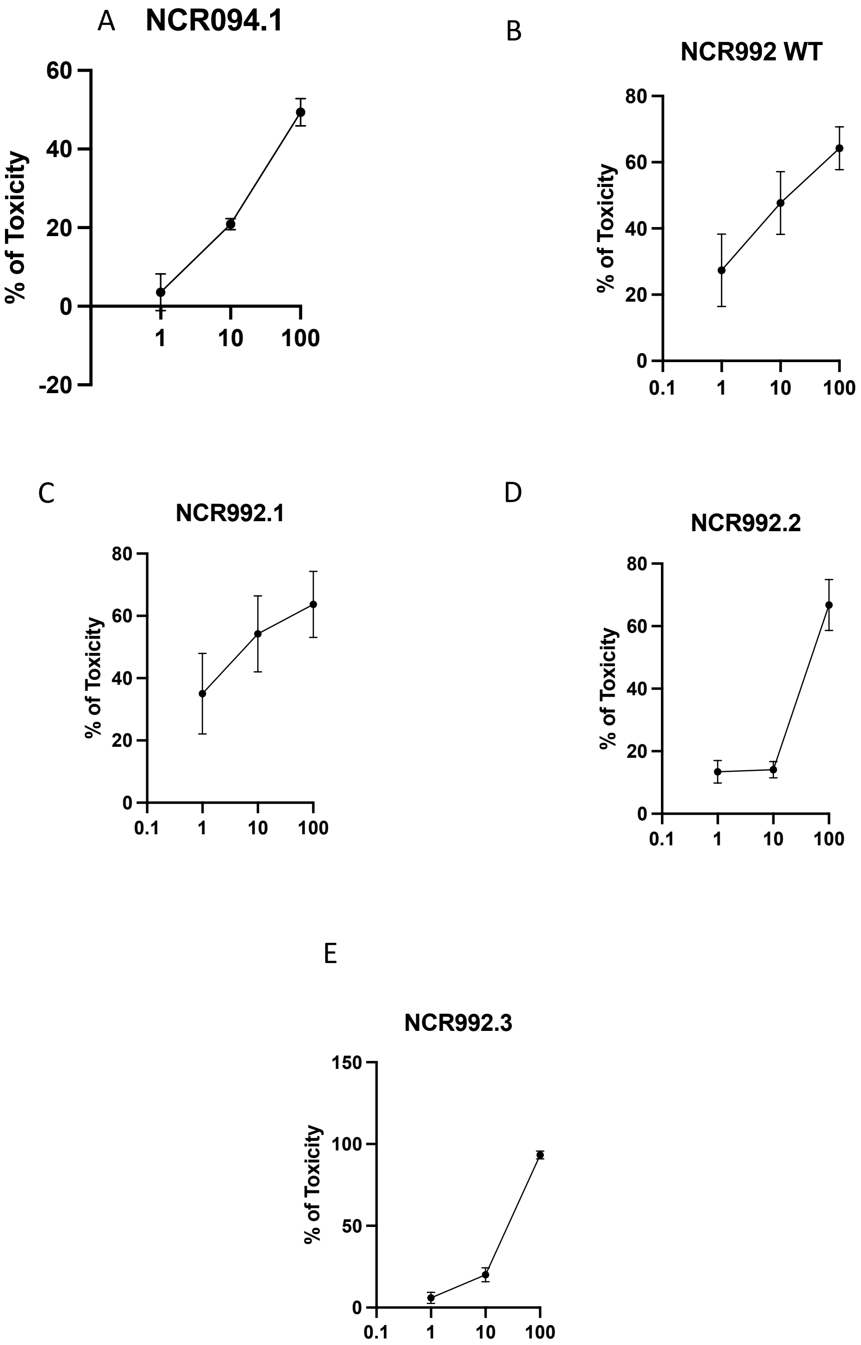

Figure S.1. Dose-dependent hemolysis assay for the NCR collection used in this study.
1% Human red blood cells were treated with 10-fold concentration (100, 10, 1, 0 µM) of NCRs **A)** (NCR094.1, **B)** NCR992 WT) **C)** NCR992.1, **D)** NCR992.2, and **E)** NCR992.1for 18 hrs without shaking. 100 µM of supernatants were transferred into new 96-well plate and the absorbance was measured using a plate reader at 405 nm wavelength. The results were represented as average of the % hemolysis and error bars were ± SD values from three independent experiments.

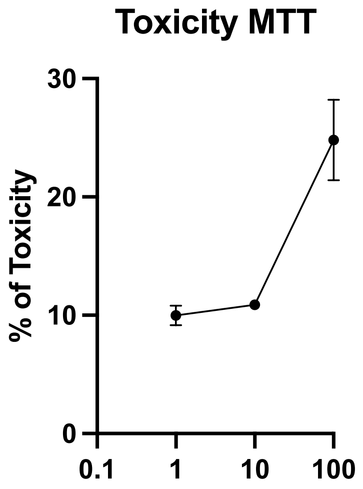

Figure S. 2. Dose-dependent toxicity assay for NCR collection used in this study toward Leukemia cell line (K562).

The cells were treated with were treated with 10-fold concentration (100, 10, 1, 0 µM) of NCR992.1 After 24 hrs exposure, MTT were added, and cells were incubated for 4 hrs. The Formazan crystals was dissolved in dimethyl sulfoxide solvent (DMSO). Absorbance was read at 540 and the toxicity% were calculated and the results were presented as means and error bars were ± SD values from four independent experiments.
